# Traumatic Floating Clavicle: Is This a Rare Injury?

**DOI:** 10.7759/cureus.7525

**Published:** 2020-04-03

**Authors:** Marios Salmas, Stavros Angelis, Dimitrios Chytas, Alexandros Apostolopoulos, Dimitrios Filippou

**Affiliations:** 1 Orthopaedics, National and Kapodistrian University of Athens Medical School, Athens, GRC; 2 Surgical Anatomy, National and Kapodistrian University of Athens Medical School, Athens, GRC; 3 Orthopaedics, Panagiotis & Aglaia Kyriakou Children's Hospital, Athens, GRC; 4 Orthopaedics, Korgialenio-Benakio Hellenic Red Cross Hospital, Athens, GRC; 5 Anatomy, European University of Cyprus, Nicosia, CYP; 6 Orthopaedics, East Surrey Hospital/Surrey and Sussex Healthcare National Health Service Trust, Redhill, GBR; 7 Surgery, National and Kapodistrian University of Athens Medical School, Athens, GRC

**Keywords:** clavicle, floating clavicle, panclavicular dislocation, bipolar clavicle dislocation, shoulder, sternoclavicular joint, acromioclavicular joint

## Abstract

Bipolar clavicle dislocation is thought to be a rare injury pattern. Even experienced orthopaedic surgeons may have not come across this entity during their careers. We report a misdiagnosed case of a 65-year-old male who underwent a motorcycle accident and was surgically treated six months post-injury. This case has been the ground for research since then. We have come to the conclusion that this type of injury is probably not so uncommon as previously thought. Careful evaluation is of immense importance during diagnosis protocol, and practitioners should be aware of this injury pattern in order to avoid misdiagnosis.

## Introduction

Floating clavicle is the injury pattern describing simultaneous ipsilateral dislocation of sternoclavicular and acromioclavicular joints [1]. The terms bifocal or bipolar clavicle dislocation and panclavicular dislocation have also been used [1,2].

The first case was reported by Porral in 1831, but for more than 90 years no other similar trauma model had been recorded [3]. It was in 1924 when Beckman reviewed and published a case series consisting of 16 bipolar clavicle dislocations, meaning that even though this type of injury had been identified, no research had been addressed upon this matter [4]. Another 58 years had to pass until a new report was presented by Gearen and Petty [5]. The writers quote: "The frequency of this injury has undoubtedly increased since Beckman's report but we have been unable to find even a case report on the lesion" [5]. Since then, single case reports and small case series (consisting mostly of two cases; except for Sanders et al. case series study) have been recorded [2,6-8]. To our knowledge, more than 40 cases in total have been reported since the early 1980s, making us question the rarity of the injury [1,2,9].

The floating clavicle is considered to be a rare entity [1,2,6-10]. We present a misdiagnosed case of a 65-year-old male who underwent a motorcycle accident and was surgically treated six months post-injury. This case has been the ground for research since then. The aim of our study is not only to present the case, its treatment and outcome, but also to review the literature and stress our theory that this injury pattern is probably not so uncommon as previously assumed. 

## Case presentation

A 65-year-old right-hand dominant male ex construction worker who was involved in a motorcycle road traffic accident was referred to the emergency department of a Level I Trauma Center. He was alert and reported right shoulder and hemithorax pain after a crushing and rolling (trunk torsion) injury under the car involved in the accident. In total, his past medical history was unremarkable, except for hypertension under treatment.

Physical examination revealed diffuse swelling, mild abrasions and pain on palpation on the anterior aspect of the shoulder region. Mild restriction of glenohumeral joint range of movement due to pain was also noticed. No other concomitant injury was observed clinically. After resuscitation according to the Advanced Trauma Life Support (ATLS) protocol as outlined by the American College of Surgeons, the patient was subjected to laboratory and imaging survey. Imaging studies included chest, shoulder and cervical spine X-rays in a sitting position. No sign of abnormality was noticed at the time. Of course, had a closer and more elaborate inspection of the imaging survey taken place, the physician would have detected a mild overlap of the acromioclavicular joint. This could have made him sceptical about the underlying lesion. After monitoring the patient for 24 hours, as the hospital's protocol dictates, he was discharged with the hand in a sling and instructions for pain management and revaluation within a week in the Orthopaedic Outpatient Department. The patient failed to attend his follow up-appointment.

**Figure 1:**
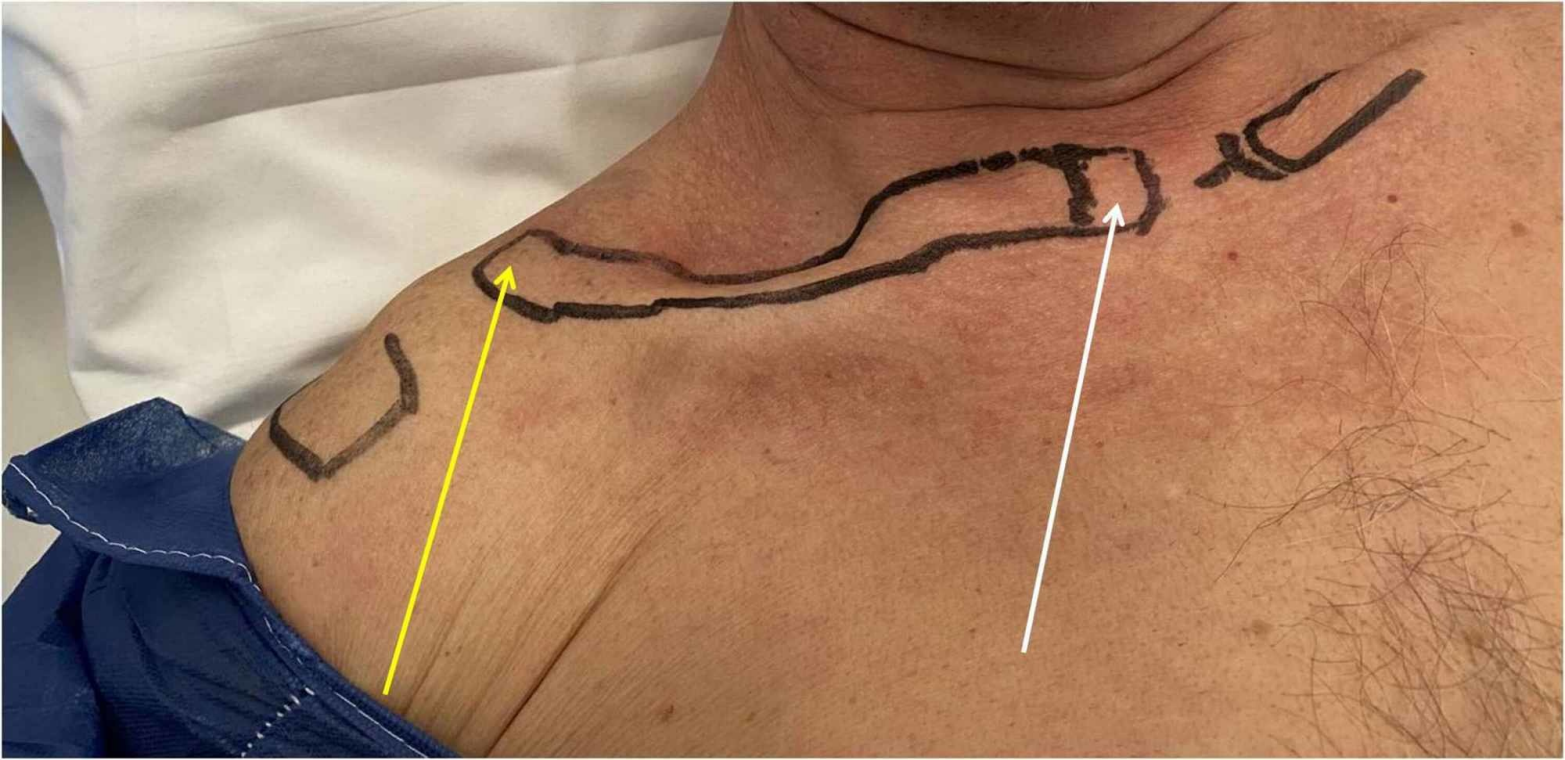
Deformity of the clavicle when lying. Posterior-superior dislocation of the sternoclavicular joint (white arrow), posterior-superior dislocation of the acromioclavicular joint (yellow arrow).

Six months post-injury, the patient remained symptomatic when visiting the Orthopaedic Outpatient Department. He had arranged an appointment on his own initiative. Swelling had subsided; gross deformity when lying and limited glenohumeral joint movements were obvious. When in a sitting or standing position, acromioclavicular deformity improved and sternoclavicular deformity deteriorated. In particular, physical examination revealed posterior-superior dislocation of the sternoclavicular joint when lying, posterior-superior dislocation of the acromioclavicular joint (type IV, according to the Rockwood classification [11]) and decreased range of motion of the right shoulder, with 45° of abduction, 80° of flexion and minimal rotation (Figure 1).

The clavicle was exceptionally mobile during palpation (Video 1). 

**Video 1:** Demonstration of the clavicle’s mobility.

**Figure 2:**
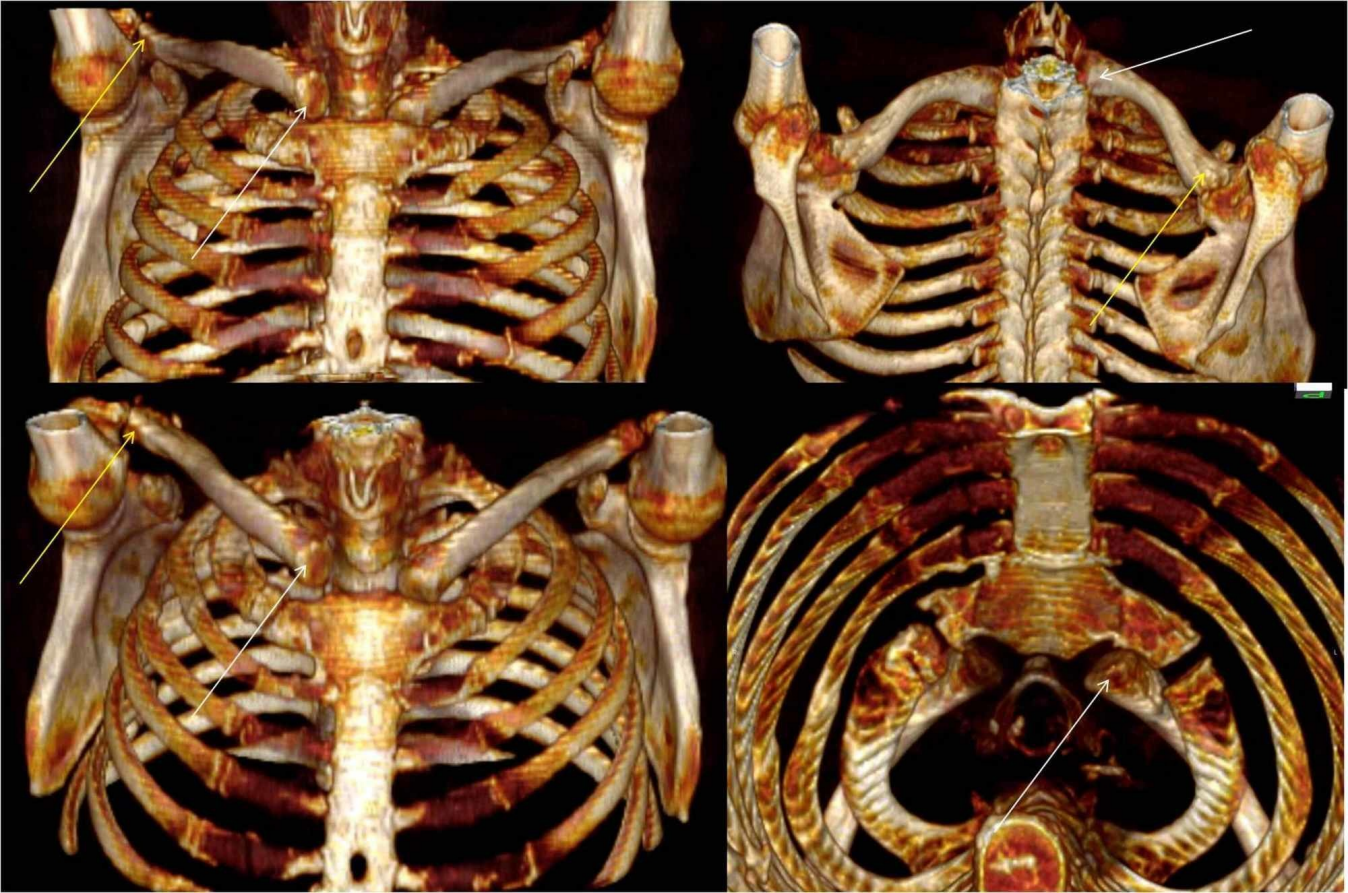
Three-dimensional computed tomography scans. The white arrows point to the posterior-superior dislocation of the sternoclavicular end. The yellow arrows point to the posterior-superior dislocation of the acromioclavicular end.

The patient also reported ongoing pain that prevented him from simple daily activities. New imaging study was performed. This included X-rays and a three-dimensional computed tomography scan (Figure 2). Surgery was scheduled during the next visit. 

**Figure 3:**
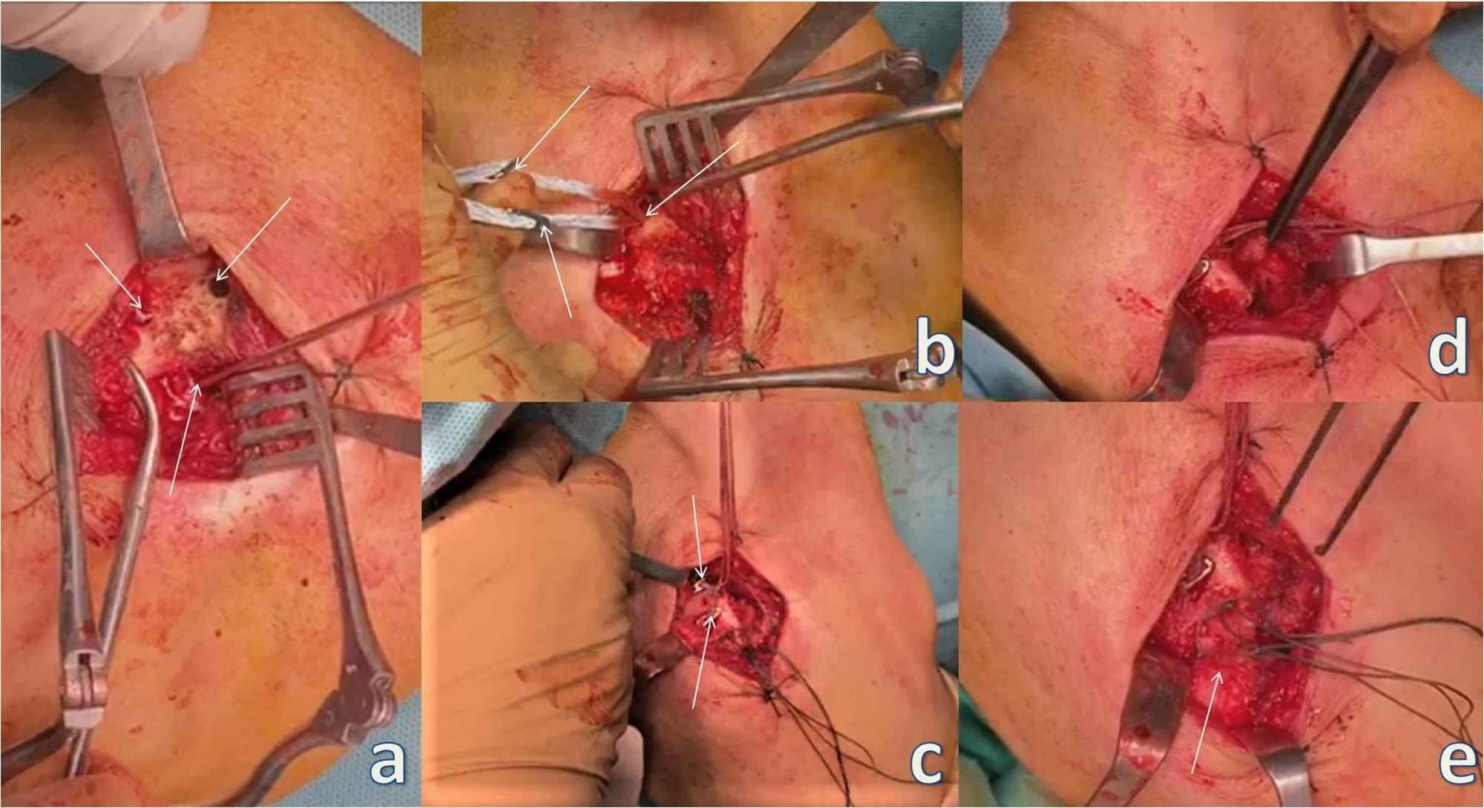
Acromioclavicular stabilization. (a) The arrows point to the two holes on the clavicle and to the one at the base of the coracoid process. (b) The arrows point to the placement of the endobutton at the base of the coracoid process and the two buttons meant for placement at the clavicle. (c) The arrows point to the two buttons placed at the clavicle. (d) Forceps holding the partial detachment of the coracoacromial ligament before placement at the inferior aspect of the distal clavicle for additional biologic stabilization. (e) The arrow points to the restored joint capsule.

The patient was placed in the beach chair position and given general anaesthesia. A 4-cm incision was performed extending from over the lateral aspect of the clavicle to over the just lateral aspect of the coracoid process. Fibrous tissue from the lateral aspect of the clavicle and the acromioclavicular joint was removed. Partial detachment of the coracoacromial ligament (CAL) was also performed. Three 3-mm holes were drilled: one at the base of the coracoid process and two at the distal end of the clavicle. A "Y Button" configuration technique was used to stabilize the acromioclavicular joint. Additionally, the partial detachment of the CAL was used for biological stabilization of the joint, since it was turned into a coracoclavicular ligament with the use of a bioabsorbable suture anchor placed at the inferior aspect of the distal clavicle. Finally, the joint capsule was restored for further stabilization (Figure 3).

**Figure 4:**
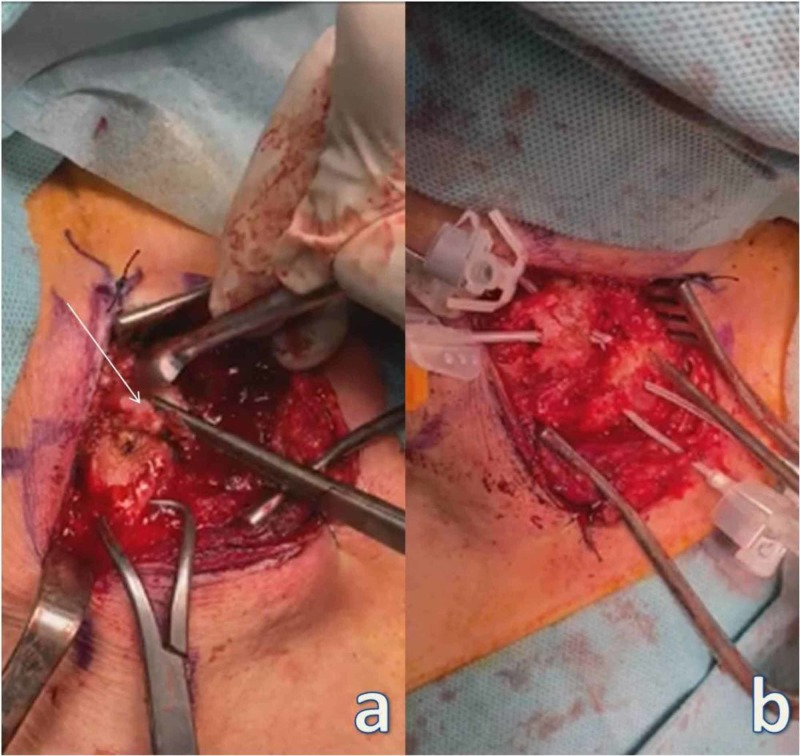
Sternoclavicular stabilization. (a) The arrow points to the articular disc being removed. (b) Two catheters placed through the drilled holes in the distal clavicle and exiting through the articular surface, and two catheters placed through the drilled holes of the manubrium of the sternum and exiting through the articular surface, respectively.

A second 6-cm curved incision was performed over the sternoclavicular joint. Fibrous tissue from the medial aspect of the clavicle and the sternoclavicular joint and the articular disc were removed. Two 3-mm holes were drilled in the medial clavicle through the articular surface. Respectively, two 3-mm holes were drilled through the articular surface of the manubrium of the sternum (Figure 4).

**Figure 5:**
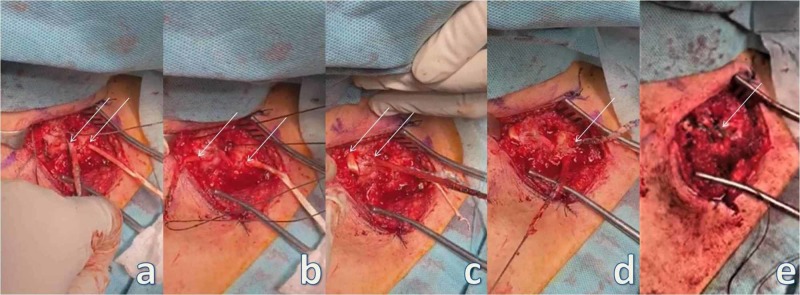
Sternoclavicular stabilization. (a-d) The arrows point to the figure-eight technique using semitendinosus autograft. (e) The arrow points to the final positioning and stabilization of the graft with sutures.

A semitendinosus autograft was used in a figure-eight technique (Figure 5). Fixation of both joints was stable during intraoperative tests. 

**Figure 6:**
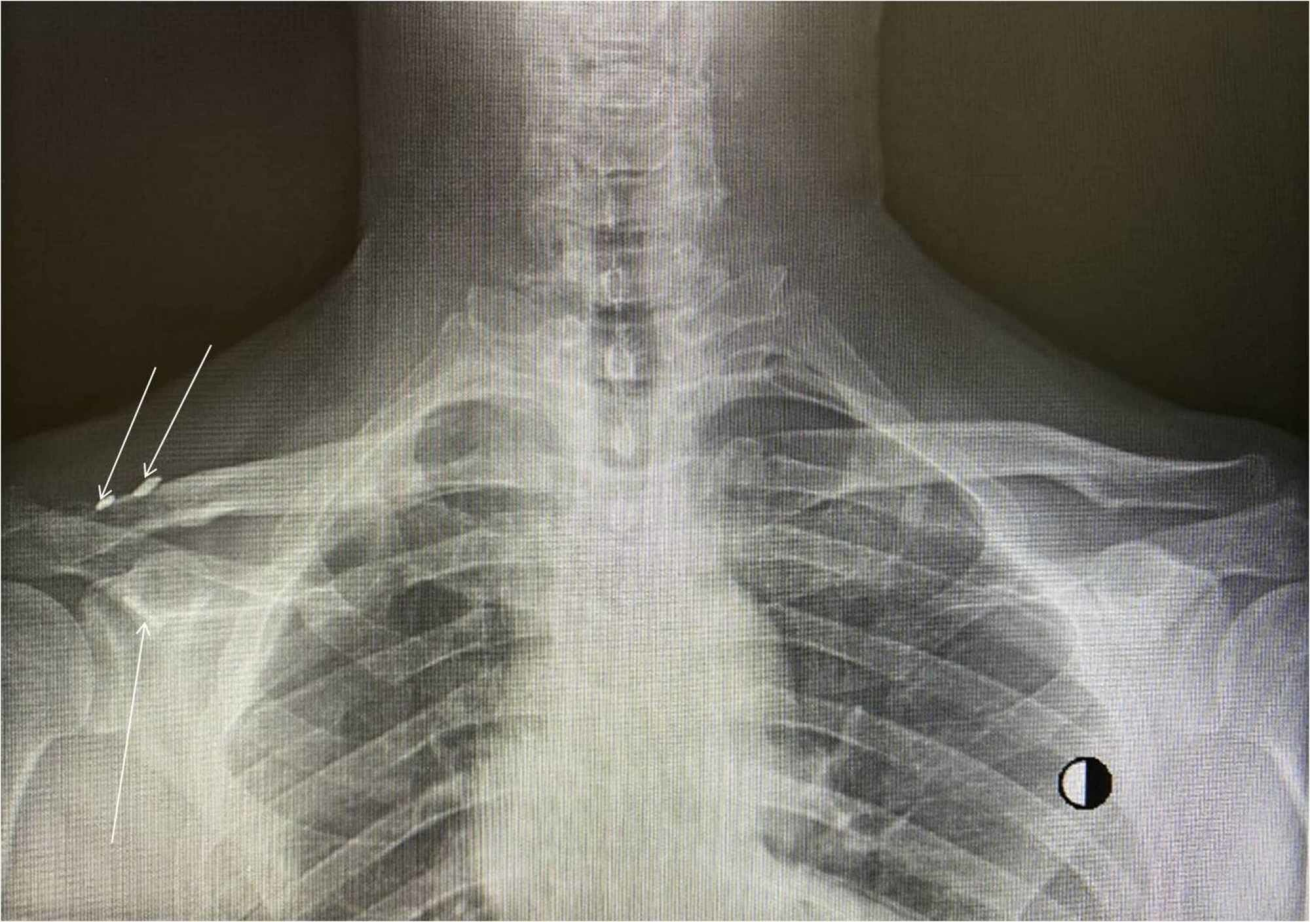
Radiograph demonstrating almost accurate reduction; however, functional results were excellent. The arrows point to the buttons of the "Y Button" configuration.

Postoperatively, the patient’s right shoulder was placed in a sling for comfort. Radiographs revealed stable fixation and almost accurate reduction (Figure 6). Passive motion exercises were initiated one week postoperatively and active strengthening exercise began six weeks post-surgery. One year post-surgery, the patient is able to perform pain-free full range of movement.

## Discussion

Bifocal clavicle injuries are usually the result of clavicle rotation around its midpoint. This ordinarily ends in a posterior dislocation of the acromioclavicular joint and anterior dislocation of the sternoclavicular joint without this being the rule [8,12]. In particular, there is usually a history of a deforming force on the lateral aspect of the shoulder or a driving force squeezing the shoulders together combined with trunk torsion [6,8,12]. Therefore, this type of injury is most commonly associated with major trauma like road traffic accidents, falls from heights or heavy objects falling on to the shoulder region. Less severe trauma is not excluded, and one should not reject this possibility [8,9,12]. Our case, despite literature data, resulted in posterior-superior dislocation of both clavicle ends, probably due to the severity of the force released upon the shoulder region. On the other hand, references were confirmed as far as the mechanism of injury is concerned. Our patient had reported trunk torsion and crushing-squeezing injury of both shoulders under the car involved in the accident.

Treatment options include conservative and surgical approaches. There is no consensus, and guidelines are not clear upon this matter [12]. Conservative treatment is a recognized and acceptable approach, especially in older, less demanding and with poor general condition patients, but also in polytrauma patients or neglected lesions [7,8,12]. There is, however, a general consensus towards the fact that conservative treatment is more prone to failure. This is advocated by the fact that most of the cases that had to be operated upon a delayed post-injury period had received some form of conservative treatment beforehand, but remained symptomatic [2]. In our case, misdiagnosis by the physician and neglection by the patient had led to an almost disabling condition that had to be addressed upon surgically.

We have noticed that most recent papers advocate surgical address on this type of injury, taking into consideration that publication bias might have existed. These reports demonstrate adequate outcomes regardless of individual characteristics in every case [2,8,12]. When floating clavicle injury is treated operatively, surgical approach to the acromioclavicular dislocation is generally supported [6]. On the other hand, there is controversy as far as the sternoclavicular dislocation is concerned [6,9]. However, as in our case, there is consensus towards the reduction of posterior injuries to the sternoclavicular joint in order to relieve or prevent from pressure on vital structures in the mediastinum [2,6]. We have chosen to operate on both joints after reviewing literature and due to the gross instability of the clavicle in our patient. We have highlighted and stressed a technique which combines a "Y Button" configuration, turning of the partial detachment of the CAL into a coracoclavicular ligament and capsule restoration for the acromioclavicular joint dislocation. For the sternoclavicular joint dislocation, we have used a "figure-eight" technique with semitendinosus autograft in order to avoid metal devices which pose a threat for devastating complications [2]. This technique was also used in order to avoid rigid bridging/fixation of the sternoclavicular joint that could compromise shoulder range of movement. In our case, imaging results probably were not optimal but functional and cosmetic results were excellent (Figure 6).

The floating clavicle is considered to be a rare entity [1,2,6-10]. It is our belief that this injury pattern is probably not so uncommon as previously estimated and cited in most literature. We have based this theory upon the fact that after its first report in 1831, and until the early 1980s, there had been only one study addressing this injury [3,4]. Since then more than 40 cases in total have been reported. This is probably be due to the high frequency of road traffic accidents during the past few decades. On the other hand, as in our case, lack of awareness amongst physicians and non-specific history and physical findings frequently lead to misdiagnosis [9,11,12]. In addition, polytrauma patients are also at greater risk of misdiagnosis [12].

## Conclusions

The floating clavicle is considered to be a rare entity. We have come to the conclusion that this type of injury is not so uncommon as previously estimated, based on literature review and our own experience. Careful evaluation and high index of suspicion are of immense importance during diagnosis protocol. Practitioners should be aware of this injury pattern in order to avoid misdiagnosis. 
